# Clinical phenotype of pulmonary vascular disease requiring treatment in extremely preterm infants

**DOI:** 10.1186/s12887-024-04943-4

**Published:** 2024-07-20

**Authors:** Ki Teak Hong, Seung Han Shin, Ee-Kyung Kim, Han-Suk Kim

**Affiliations:** 1 Department of Pediatrics, Seoul National University Children’s Hospital, Seoul, Republic of Korea; 2 Department of Pediatrics, Seoul National University College of Medicine, 101 Daehak-ro, Jongno-gu, Seoul, 03080 Republic of Korea

**Keywords:** Pulmonary hypertension, Extremely preterm infant, Persistent pulmonary hypertension of newborn, Bronchopulmonary dysplasia, Neonatal intensive care units

## Abstract

**Background:**

Pulmonary vascular disease (PVD) and pulmonary hypertension (PH) is a significant disorder affecting prognosis of extremely preterm infants. However, there is still a lack of a consensus on the definition and optimal treatments of PH, and there is also a lack of research comparing these conditions with persistent pulmonary hypertension of newborn (PPHN), early PH, and late PH. To investigate PH in extremely preterm infants, this study compared the baseline characteristics, short-term outcomes, and treatment duration, categorized by the timing of requiring PH treatment.

**Methods:**

This study retrospectively analyzed extremely preterm infants admitted to a single tertiary center. Between 2018 and 2022, infants with clinical or echocardiographic diagnosis of PH who required treatment were divided into three groups based on the timing of treatment initiation: initial 3 days (extremely early-period), from day 4 to day 27 (early-period), and after day 28 (late-period). The study compared the outcomes, including mortality rates, bronchopulmonary dysplasia (BPD) severity, PH treatment duration, and oxygen therapy duration, among the three groups.

**Results:**

Among the 157 infants, 67 (42.7%) were treated for PH during their stay. Of these, 39 (57.3%) were treatment in extremely early, 21 (31.3%) in early, and seven (11.4%) in late periods. No significant differences were observed in maternal factors, neonatal factors, or morbidity between the three groups. However, infants who received extremely early-period treatment had a higher mortality rate, but shorter duration of noninvasive respiratory support, oxygen therapy, and PH medication use. On the other hand, the late-period treatment group received longer durations of respiratory support and treatment.

**Conclusions:**

This study revealed differences in mortality rates, respiratory outcomes, and treatment duration between the three groups, suggesting varying pathophysiologies over time in extremely preterm infants.

**Supplementary Information:**

The online version contains supplementary material available at 10.1186/s12887-024-04943-4.

## Background

The pulmonary vasculature plays a critical role in facilitating gas exchange in the lungs. During fetal development, the pulmonary vasculature undergoes intricate changes in preparation for the transition from intrauterine to extrauterine life [1]. However, certain factors, such as prematurity, congenital heart defects, or prenatal insults, can disrupt this process, leading to pulmonary vascular disease (PVD) in newborns [2–5].

Early PVD in newborns refers to abnormalities in the development and function of pulmonary vasculature, which can have significant clinical implications. Understanding the relationship between pulmonary vascular development and neonatal lung disease is crucial to understand its clinical significance. In neonatal lung diseases, abnormalities in pulmonary vascular development can exacerbate conditions such as persistent pulmonary hypertension of the newborn (PPHN), bronchopulmonary dysplasia (BPD), and respiratory distress syndrome (RDS) [1, 6–8]. For example, in PPHN, elevated pulmonary vascular resistance impedes blood flow through the lungs, resulting in hypoxemia and respiratory distress. Similarly, impaired pulmonary vascular development contributes to abnormal lung growth and function in BPD, exacerbating respiratory compromise.

The clinical significance of early PVD in newborns is its potential to increase the respiratory morbidity and mortality rates. Infants with compromised pulmonary vascular function often require intensive respiratory support, including mechanical ventilation and pharmacological interventions, to optimize oxygenation and pulmonary blood flow [9, 10]. Moreover, long-term sequelae such as pulmonary hypertension (PH) and chronic respiratory insufficiency may ensue, further impacting the infant's health and development [11–15].

However, there are differences in the definitions of PVD and PH phenotypes among studies owing to a lack of consensus. Additionally, studies comparing baseline characteristics and prognosis across PH phenotypes are lacking. The results of echocardiography may vary depending on the infant’s condition and the operator. Therefore, analysis of the characteristics and phenotypes based on treatment status and timing is helpful in understanding the pathophysiology of PVD. Thus, this study aimed to describe the baseline characteristics and compare short-term respiratory outcomes in extremely preterm infants based on the timing of requiring PH treatment.

## Methods

This retrospective study included extremely preterm infants (gestational age[GA] < 28 weeks) admitted to the neonatal intensive care unit (NICU) of Seoul National University Children’s Hospital in Republic of Korea between January 2018 and December 2022. Infants with cardiac anomalies (except for patent foramen ovale, small atrial septal defect, small ventricular septal defect, and patent ductus arteriosus [PDA]) and those treated with inhaled nitric oxide(iNO) without clinical diagnosis or echocardiographic diagnosis of PH in their medical records were excluded. Clinical diagnosis of PPHN or PH with PDA was defined as differential desaturation (a difference of more than 10% between pre- and post-ductal oxygen saturation [SpO_2_]) accompanied by severe hypoxemia requiring a high fraction of inspired oxygen (FiO_2_). In patients without PDA, particularly in the late group, PH was suspected based on symptoms of recurrent hypoxemia, prompting echocardiography. Echocardiographic diagnosis included at least one or more of the following: (1) peak velocity of tricuspid regurgitation (estimated right ventricular systolic pressure) ≥ 2.8 m/s, (2) any cardiac shunt flow with bidirectional or right-to-left flow, (3) continuous left-to-right shunting through a PDA with a maximum velocity < 2.0 m/s, or (4) every degree of interventricular septal flattening [11]. Echocardiography was performed by neonatologists, pediatric cardiologists, and fellows in neonatology or pediatric cardiology. If the examination was conducted by a fellow, the results were confirmed by a neonatologist or pediatric cardiologist. All infants were cared for by NICU clinical teams in accordance with local guidelines. The clinical teams aimed for the SpO_2_ to be as close to 90% as possible (lower alarm limit of 85%). If PH was suspected (clinically or echocardiographically) in infants, the target SpO_2_ was as high as 95%. According to target SpO_2_, FiO_2_ was adjusted by the clinical teams. If there was diagnosis of PH requiring a FiO_2_ ≥ 0.6–0.7, treatment initiation for PH, such as iNO or sildenafil, was considered.

In this study, extremely early-period treatment was defined as initial treatment within initial 3 days with clinical or echocardiographic diagnosis of PH. Early-period treatment was defined as initial treatment between 4 and 27 days of age with clinical or echocardiographic diagnosis of PH. Late-period treatment was defined as initial treatment after 28 days of age with clinical or echocardiographic diagnosis of PH.

Data on maternal, neonatal, and postnatal characteristics were collected through an electronic medical record review. Oligohydramnios was defined as an amniotic fluid index of less than 5, or if the amniotic fluid index value was not recorded, it was determined based on the obstetrician’s opinion during prenatal visits. Regardless of the presence of proteinuria or edema, maternal hypertension, defined as pregnancy-induced hypertension (PIH) or chronic hypertension (Chronic HTN), was determined based on neonatal medical records. Eclampsia and preeclampsia were classified as PIH. However, if pre-existing hypertension worsened, it was defined as chronic HTN. Intraventricular hemorrhage was defined as grade ≥ 3 on the Papile grading system on brain ultrasonography. Early onset sepsis was defined as a positive result in blood culture obtained within the first 7 days of life, and positive blood culture at any other period was defined as late-onset sepsis [16]. Necrotizing enterocolitis was defined as a modified Bell’s criteria of grade 2 or higher [17]. Data on echocardiographically moderate-to-large PDA were collected, and were graded based on previous studies [18]. The duration of admission was defined as the length of the hospital stay following admission to the NICU. BPD diagnosis and severity were assessed at 36 weeks’ postmenstrual age in accordance with the 2019 Jensen definition [19]. Duration of invasive ventilation was defined as the period during which the patient is intubated and utilizing conventional ventilation, high frequency oscillatory ventilation or neurally adjusted ventilatory assist. Duration of noninvasive ventilation was defined as the period during which the patient is extubated and utilizing noninvasive positive pressure ventilation, non-invasive ventilation with neurally adjusted ventilatory assist, bilevel positive airway pressure, nasal continuous positive airway pressure, and high flow nasal cannula. Duration of supplemental oxygen was defined as the period during which the patient was exposed to additional oxygen (FiO_2_ > 0.21) with or without respiratory support. Duration of PH medication was defined as the period during which the patient was treated with PH medications, including iNO, sildenafil, iloprost, bosentan, milrinone and treprostinil. To minimize selection bias, deaths were excluded when comparing respiratory outcomes.

Data are presented as mean ± standard deviation (SD), median (interquartile range), or frequency with percentage. Maternal, neonatal, and postnatal continuous variables were compared between infants with and without PH, and between the various initiation timings of PH treatment, using Student’s t-test, Welch’s t-test, Mann–Whitney U test, ANOVA, or the Kruskal–Wallis test. Proportions were analyzed using the Fisher’s exact test. A Kaplan–Meier curve was plotted to compare the duration of supplemental oxygen therapy and PH medication according to patient group. Differences between groups were assessed using the log-rank test. Statistical significance was set at *P* < 0.05. Data analysis was performed using R version 4.3.1 (R Foundation for Statistical Computing, Vienna, Austria) and GraphPad Prism version 10.1.1 for Mac (GraphPad Software, Boston, MA, United States).

The study protocol was reviewed and approved by the Institutional Review Board of Seoul National University Hospital (IRB No.: 2310–128-1478; approval date: November 06, 2023). The study was conducted in accordance with the principles stated in the Declaration of Helsinki.

## Results

**Fig. 1:**
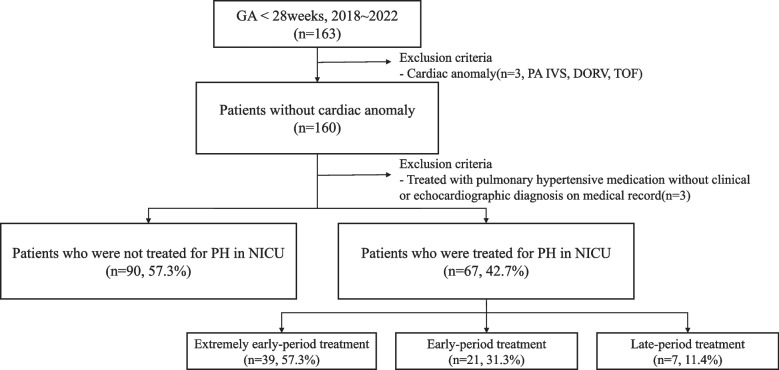
Flowchart of patients treated for pulmonary hypertension. DORV, double-outlet right ventricle; GA, gestational age; NICU, neonatal intensive care unit; PA IVS, pulmonary atresia with intact ventricular septum; TOF, Tetralogy of Fallot

A total of 163 eligible infants were born between January 2018 and December 2022, of whom 157 were included because 3 infants with cardiac anomalies and 3 infants treated for acute respiratory failure without clinical or echocardiographic evidence of PH were excluded. Sixty-seven infants (42.7%) were diagnosed with PH and were treated with medication. Based on the definitions in this study, 39 infants (57.3%) were classified as extremely early-period treatment, 21 (31.3%) as early-period treatment, and seven (11.4%) as late-period treatment (Fig. 1).

The median duration of premature rupture of membranes (PROM) was 23.0 days in the group treated for PH compared to 6 days in the untreated group, indicating a significant difference. Infants treated for PH had a lower incidence of chorioamnionitis (64.8% vs. 46.3%). Neonatal characteristics, including GA (26.1 weeks vs. 25.2 weeks), birth body weight (831 g vs. 706.3 g), 1-min (3[2;5] vs. 3[1;4]) and 5-min (7[5;7] vs. 6[4;7]) Apgar scores were significantly different between the treated and untreated groups. Regarding morbidity, significant differences were observed between the groups in terms of respiratory distress syndrome (85.6% vs. 98.5%), air leak (2.2% vs. 19.4%), intraventricular hemorrhage (8.0% vs. 22.6%), and early onset sepsis (6.7% vs. 19.4%). However, maternal characteristics, including maternal age, oligohydramnios, antenatal corticosteroid use, caesarean section, maternal diabetes mellitus, and maternal hypertensive disease showed no significant differences between the treated and untreated groups for PH (Supplemental Table 1).

Death at discharge (4.4% vs. 43.3%), duration of admission (96.5 days vs. 133.5 days), BPD severity, duration of invasive ventilation (17.5 days vs. 45.5 days), and duration of supplemental oxygen therapy (75.5 days vs. 157.5 days) were significantly different between the treated and untreated infants for PH (Supplemental Table 2).

When infants who received extremely early-, early-, and late-period treatments were compared, no significant differences were observed in maternal characteristics, neonatal characteristics, or neonatal morbidity, except for oligohydramnios (38.5% vs. 14.3% vs. 57.1%) (Table 1).

**Table 1:** Baseline characteristics of patients according to the initial treatment period for pulmonary hypertension

	Extremely early-period treatment (*n* = 39)	Early-period treatment (*n* = 21)	Late-period treatment (*n* = 7)	*P*
**Maternal factors**				
Maternal age, y	34.7 ± 3.4	34.0 ± 2.5	33.3 ± 2.7	0.445
Oligohydramnios, n (%)	15 (38.5%)	3 (14.3%)†	4 (57.1%)†	**0.045**
PROM, n (%)	18 (46.2%)	8 (38.1%)	4 (57.1%)	0.632
PROM duration, d	31.2 ± 24.1	16.5 ± 18.6	20.0 ± 18.7	0.278
Chorioamnionitis, n (%)	17 (43.6%)	9 (42.9%)	5 (71.4%)	0.445
Antenatal corticosteroids, n (%)	38 (97.4%)	18 (85.7%)	7 (100%)	0.139
Cesarean section, n (%)	22 (56.4%)	7 (33.3%)	3 (42.9%)	0.227
Maternal DM, n (%)	GDM 4 (10.3%) Overt DM 1 (2.6%)	GDM 0 (0%) Overt DM 1 (4.8%)	GDM 2 (28.6%) Overt DM 0 (0%)	0.141
Maternal hypertensive disorder, n (%)	PIH 2 (5.1%) Chronic HTN 3 (7.7%)	PIH 2 (9.5%) Chronic HTN 2 (9.5%)	PIH 2 (28.6%) Chronic HTN 0 (0.0%)	0.379
**Neonatal factor**				
GA, wk	25.2 ± 1.4	25.0 ± 1.3	25.5 ± 1.5	0.623
Body weight, g	729.5 ± 207.7	672.4 ± 162.4	678.6 ± 136.2	0.496
Small for gestational age, n (%)	7 (17.9%)	2 (9.5%)	1 (14.3%)	0.780
Multiple gestation, n (%)	Singleton 9 (23.1%) Twin 24 (61.5%) Triplet 6 (15.4%)	Singleton 7 (33.3%) Twin 11 (52.4%) Triplet 3 (14.3%)	Singleton 4 (57.1%) Twin 2 (28.6%) Triplet 1 (14.3%)	0.430
Apgar score 1 min	2 [1.0; 4.0]	3 [2.0; 4.0]	2.0 [1.0; 2.5]	0.078
Apgar score 5 min	5 [3.5; 6.0]	6 [5.0; 7.0]	4.0 [4.0; 6.5]	0.134
**Other outcomes, n(%)**				
Respiratory distress syndrome, n (%)	39 (100.0%)	20 (95.2%)	7 (100.0%)	0.418
Air leak, n (%)	9 (23.1%)	2 (9.5%)	2 (28.6%)	0.387
Pulmonary hemorrhage, n (%)	9 (23.1%)	1 (4.8%)	0 (0.0%)	0.135
Neonatal seizure, n (%)	5 (12.8%)	1 (4.8%)	1 (14.3%)	0.498
Intraventricular hemorrhage, n (%)	10 (28.6%)	2 (10.0%)	2 (28.6%)	0.224
Congenital infection, n (%)	2 (5.1%)	0 (0.0%)	0 (0.0%)	0.630
Early onset sepsis, n (%)	10 (25.6%)	2 (9.5%)	1 (14.3%)	0.387
Late onset sepsis, n (%)	4 (10.3%)	6 (28.6%)	2 (28.6%)	0.128
Necrotizing enterocolitis, n (%)	1 (2.6%)	2 (9.5%)	0 (0.0%)	0.484
Spontaneous intestinal perforation, n (%)	3 (7.7%)	4 (19.0%)	1 (14.3%)	0.381
Surgical necrotizing enterocolitis, n (%)	1 (2.6%)	2 (9.5%)	0 (0.0%)	0.484
Surgical retinopathy of prematurity, n (%)	17 (43.6%)	12 (57.1%)	5 (71.4%)	0.320
Moderate to large PDA, n (%)	26 (66.7%)	15 (71.4%)	5 (71.4%)	0.926
PDA treatment, n (%)	16 (41.0%)	14 (66.7%)	5 (71.4%)	0.102
PDA ligation operation, n (%)	8 (20.5%)	10 (47.6%)	3 (42.9%)	0.077

Data are expressed as numbers (%), median [interquartile range] for using nonparametric method, or mean ± SD for using parametric method. †*P*-value < 0.05 in the post-hoc analysis

*Chronic HTN* chronic hypertension, *DM* diabetes mellitus, *GA* gestational age, *GDM* gestational diabetes mellitus, *PDA* patent ductus arteriosus, *PIH* pregnancy-induced hypertension, *PROM* premature rupture of membranes

Fisher’s exact test was performed to describe differences. ANOVA was performed to describe difference for parametric analysis, and Kruskal–Wallis rank sum test was performed to describe difference for non-parametric analysis

Mortality was significantly different between infants provided with extremely early- and late- period treatment in the post-hoc analysis (53.8% vs. 0.0%). More than half of the infants who were treated in extremely early period died, and all of them died within the first 28 days of life. Infants with extremely early-period treatment had a shorter duration of non-invasive ventilation (37.9 days vs. 84.0 days), supplemental oxygen (122.5 days vs. 242.0 days), and PH treatment (5.0 days vs. 182.0 days) compared to infants with late-period treatment (Table 2 and Supplemental Table 3). Significant differences were observed in the frequencies of iNO, sildenafil, and bosentan use between the groups (Table 3).

**Table 2:** Outcomes of patients according to initial treatment period for pulmonary hypertension

	Extremely early-period treatment (*n* = 39)	Early-period treatment (*n* = 21)	Late-period treatment (*n* = 7)	*P*
Death at discharge, n (%)	21 (53.8%)†	8 (38.1%)	0 (0.0%)†	**0.019**
Timing of death event. n (%)	PND ≤ 3 days 15 (71.4%) PND ≤ 14 days 18 (85.7%) PND ≤ 28 days 21 (100%)	PND ≤ 14 days 4 (50.0%) PND ≤ 28 days 5 (62.5%) PND ≤ 60 days 8 (100%)		
Duration of admission*, d	104.0 [95.0;161.0]	134.0 [114.0;142.0]	163.0 [135.5;197.0]	0.139
BPD severity (2019 NICHD-NRN Jensen)*, n (%)	No BPD 6 (33.3%) Grade 1 1 (5.6%) Grade 2 8 (44.4%) Grade 3 3 (16.7%)	No BPD 1 (7.7%) Grade 1 1 (7.7%) Grade 2 8 (61.5%) Grade 3 3 (23.1%)	No BPD 0 (0%) Grade 1 0 (0%) Grade 2 4 (57.1%) Grade 3 3 (42.9%)	0.383
Duration of invasive ventilation*, d	38.5 [20.0;58.0]	58.0 [41.0;67.0]	46.0 [26.0;113.5]	0.238
Duration of noninvasive ventilation*, d	37.9 ± 22.4†	47.2 ± 28.1	84.0 ± 41.4†	**0.003**
Duration of supplemental oxygen*, d	122.5 [79.0;164.0]†	189.0 [133.0;289.0]	242.0 [207.0;336.5]†	**0.014**
Duration of PH medication*, d	5.0 [2.0;29.0]†	62.0[29.0;100.0]	182.0[151.0;342.0]†	**0.002**

Data are expressed as numbers (%), median [interquartile range] for using nonparametric method, or mean ± SD for using parametric method. * Except for infants who died at discharge. †*P*-value < 0.05 in the post-hoc analysis

*BPD* bronchopulmonary dysplasia, *NICHD* Eunice Kennedy Shriver National Institute of Child Health and Human Development, *NRN* Neonatal Research Network, *PH* pulmonary hypertension, *PND* postnatal day

Fisher’s exact test was performed to describe differences. ANOVA was performed to describe difference for parametric analysis, and Kruskal–Wallis rank sum test was performed to describe difference for non-parametric analysis

**Table 3:** Pulmonary hypertension medication according to initial treatment period

	Extremely early-period treatment (*n* = 39)	Early-period treatment (*n* = 21)	Late-period treatment (*n* = 7)	*P*
iNO, n (%)	39 (100.0%)†	21 (100.0%)‡	4 (57.1%)†‡	**0.000**
Sildenafil, n (%)	3 (7.7%)†§	12 (57.1%)†‡	7 (100%)‡§	**0.000**
Iloprost, n (%)	3 (7.7%)	4 (19.0%)	2 (28.6%)	0.125
Bosentan, n (%)	1 (2.6%)†	2 (9.5%)	3 (42.9%)†	**0.007**
Milrinone, n (%)	2 (5.1%)	1 (4.8%)	0 (0.0%)	1.000
Treprostinil, n (%)	6 (15.4%)	3 (14.3%)	3 (42.9%)	0.215

Data are expressed as numbers (%). †, ‡, § *P*-value < 0.05 in the post-hoc analysis

*iNO* inhaled nitric oxide

Fisher’s exact test was performed to describe differences

**Fig. 2:**
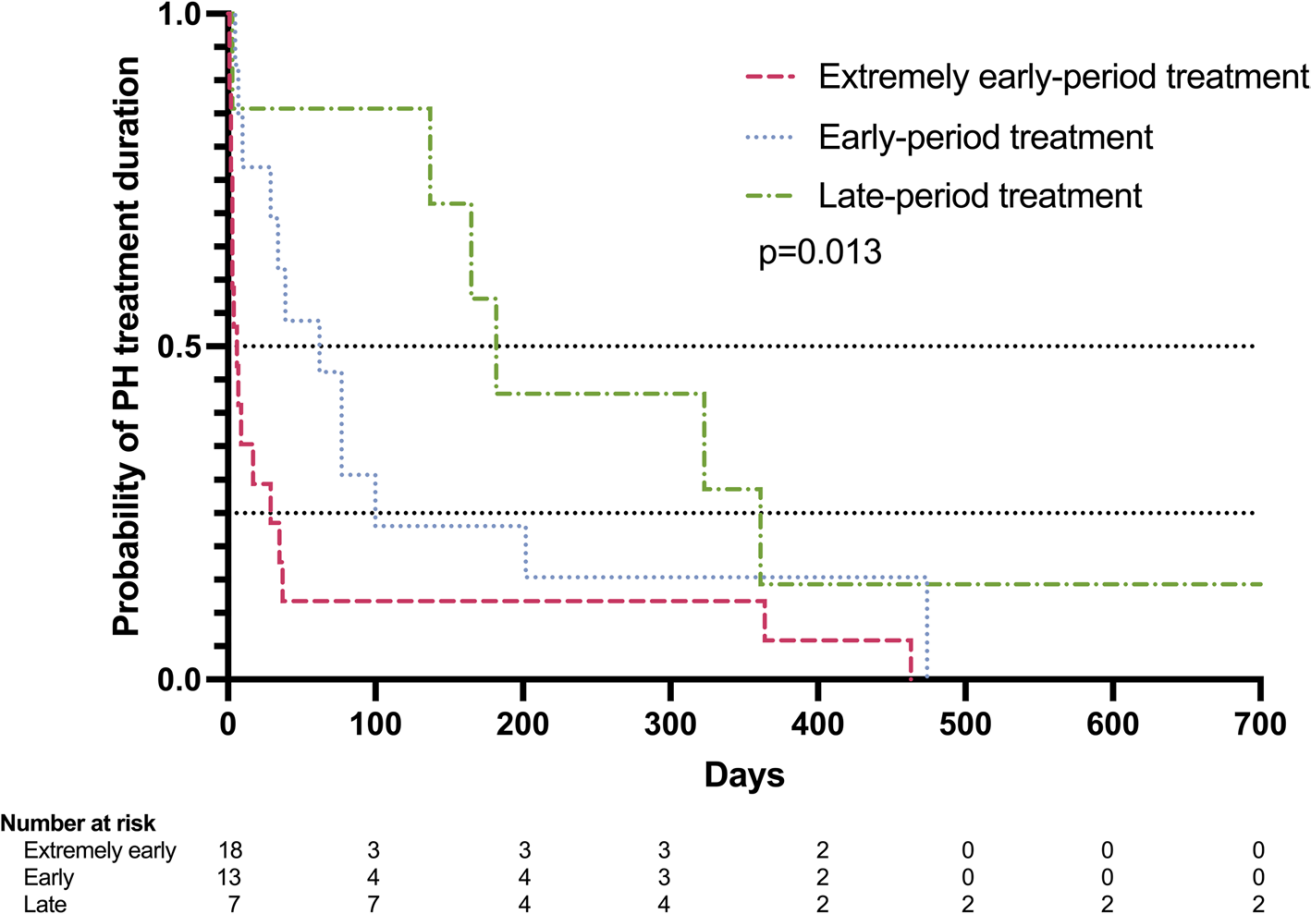
Kaplan–Meier curve of pulmonary hypertension treatment duration by initial treatment period. PH, pulmonary hypertension. A log-rank test was performed to determine differences

The Kaplan–Meier curve indicated that the duration of supplemental oxygen use and PH medication tended to be longer in the following order: no treatment for PH, extremely early-period treatment, early-period treatment, and late-period treatment (Fig. 2 and Supplemental Fig. 1).

## Discussion

As the survival rate of extremely preterm infants has increased, the recognition of PVD has also increased. During lung development in the fetal period and after preterm birth, signaling pathways, such as vascular endothelial growth factor and hypoxia-inducible factor, are affected by antenatal and postnatal factors, and lung angiogenesis is impaired [8, 20–22]. Pulmonary vascular injury presents as hemodynamic, functional, and structural abnormalities. Several studies have aimed to define the phenotypes of PVD [8, 11, 23]. In the present study, the overall incidence of PH treatment was 43% and most infants were treated in extremely early period (57.3%), followed by early period (31.3%) and late period (11.4%). In the PH treatment group, the GA and birth weight were significantly lower than those in the non-PH treatment group, and differences were observed in factors such as PROM duration and the coincidence of chorioamnionitis (Supplemental Table 1). In comparisons among the three groups based on the timing of PH treatment, except for the incidence of oligohydramnios, no differences were observed in maternal or neonatal factors (Table 1); however, significant differences were noted in mortality rates, respiratory outcomes, and duration of PH medication use among the three groups (Table 2). This suggests that different pathophysiologies may contribute to different times when the symptoms of PH manifest, indicating the need for different diagnostic and treatment approaches.

Most infants who received treatment in an extremely early period died within the first month of life. However, those who survived in this group had better respiratory outcomes than those in the other groups. Although there are variations in the definition of PPHN, previous studies have demonstrated high mortality rates. A cohort study by Nakanishi et al. showed that PPHN mortality increased to 30.5% at the time of discharge compared to 9.6% in infants without PPHN [5]. A prospective study reported a 42% mortality rate among infants with PPHN [11]. Regarding the mortality among infants with PPHN, all infants died within 28 days of birth, and 71.4% died within the first 3 days of life. However, infants with extremely early-period treatment had a significantly shorter duration of admission, noninvasive ventilation, supplemental oxygen, and PH medication than the other groups. They also showed a lower BPD severity than the other groups, although the difference was not statistically significant. Based on the previous studies and the clinical course presented in this study, it is suggested that a portion of the extremely early-period treatment group may be an acute event within the first 72 h of life and may have less impact on long-term prognosis in cases that respond to appropriate treatment with iNO and show clinical improvement in a short duration. On the other hand, in some cases in the extremely early-period treatment group, irreversible pulmonary vascular abnormalities, such as pulmonary hypoplasia, may exist, which are thought to originate from intrauterine life and do not respond to treatment. This may be a significant cause of the high mortality rate observed in this group.

Recently, the importance of PH and related PVD has been increasingly recognized as a major determinant of outcomes after preterm birth. This is a clinically significant issue, particularly as the development of pulmonary vasculature is closely related to alveolar formation, suggesting an association between the exacerbation of BPD and chronic respiratory insufficiency [14]. Initially, attention was focused on the association between PH and BPD based on the presence or absence of PH at 4 weeks of age; however, recently, there has been growing interest in the clinical significance of early PVD, as many cases manifest PH before 4 weeks of age [8, 11]. This study revealed that 76.9% (10/13 infants) of the early-period treatment group continued treatment for more than 30 days (Fig. 2), indicating that symptoms of PH persisted until the time of BPD diagnosis in the majority of cases. Although comparison among the three groups based on the timing of treatment showed no differences in maternal or neonatal factors in the present study, a recent meta-analysis indicated that oligohydramnios and small-for-gestational-age are risk factors for early PH [13]. The early-period treatment group had a higher mortality rate than the late-period and without PH treatment groups, which is also consistent with the results of a recent meta-analysis. Whether timely diagnosis and appropriate treatment intervention for systematic early PVD can improve mortality rates and respiratory morbidity is a highly intriguing clinical concern.

The late-period treatment group comprised the smallest portion among the three groups but had unique features. While no deaths were observed, the durations of mechanical ventilation, oxygen supplementation, and PH treatment were all significantly longer than those in the other groups. In addition, the frequency of maternal oligohydramnios was significantly higher. Many factors involved in the development and function of endothelial cells, such as vascular endothelial growth factor and hypoxia-inducible factor, act through signal transduction [22, 24]. High oxygen levels and pressure are believed to disrupt these signaling pathways, potentially inhibiting processes like vascular neogenesis and formation [25]. Previously, we found that pulmonary arterial hypertension group consistently underwent more aggressive respiratory management than the non-pulmonary arterial hypertension and no/mild BPD groups [26]. This suggested that aggressive respiratory management in the early-postnatal period could be a risk factor for PH. Similar pathological mechanisms may also apply to the late-period treatment group. Furthermore, while oligohydramnios has been reported as a risk factor for PH, its high frequency in the late-period treatment group is intriguing [13, 27]. Whether oligohydramnios directly contributes to the pathogenesis of PVD or indirectly through increasing the need for postnatal respiratory assistance warrants further intervention. As more than half of the late-period treatment group required medication for over six months (Fig. 2), it is important to conduct precise studies on the pathogenesis of chronic PH in the late-period treatment group.

Regarding the drugs used for intervention, iNO was administered in all patients in the extremely early- and early-period treatment groups. Sildenafil was used in all patients in the late-period treatment group, followed by frequent use in the early-period treatment group. This is presumed to be due to the urgency of symptom onset. However, its effectiveness could not be evaluated in this retrospective study. In future, well-designed prospective studies using systematic diagnostic methods and drug intervention guidelines are required to determine the effectiveness of PH treatment.

This study was limited by its retrospective nature because of missing data or incomplete follow-up. The small number of infants analyzed in this single-center study was underpowered to adequately compare the true differences between the groups. Although our center follows general practices for managing PH, there is no standardized protocol. Therefore, treatment initiation decisions and timing may vary for each infant based on the physician’s discretion.

## Conclusion

The comparison between PH treatment timing groups revealed differences in mortality rates, respiratory outcomes, and treatment duration, suggesting varying pathophysiologies over time. Infants treated in the extremely early period had better short-term respiratory outcomes; however, the mortality rates remained high, indicating potential acute events within the first 72 h of life. The majority of patients in the early-period treatment group continued PH treatment until the time of BPD diagnosis, suggesting an association between relatively high mortality rates and lung morbidity. The late-period treatment group had unique features with longer durations of respiratory support and treatment. Oligohydramnios was more frequent in this group, raising questions regarding its role in the pathogenesis of PVD. Further research on chronic PH pathogenesis in this group is crucial, as over half of the patients required treatment for more than 6 months. Future studies should focus on evaluating the effectiveness of interventions by using systematic diagnostic methods and drug intervention guidelines.

### Supplementary Information

Supplementary Material 1.

## Data Availability

The datasets generated and analyzed are not publicly available but are available from the corresponding author upon reasonable request.

## Abbreviations

BPD: Bronchopulmonary dysplasia
Chronic HTN: Chronic hypertension
DM: Diabetes mellitus
NICHD: Eunice Kennedy Shriver National Institute of Child Health and Human Development
FiO_2_: Fraction of inspired oxygen
GA: Gestational age
GDM: Gestational diabetes mellitus
iNO: Inhaled nitric oxide
NICU: Neonatal intensive care unit
NRN: Neonatal Research Network
SpO_2_: Oxygen saturation
PDA: Patent ductus arteriosus
PPHN: Persistent pulmonary hypertension of the newborn
PND: Postnatal day
PIH: Pregnancy-induced hypertension
PROM: Premature rupture of membranes
PH: Pulmonary hypertension
PVD: Pulmonary vascular disease
RDS: Respiratory distress syndrome
