# Supplementary material for: Clinical phenotype of pulmonary vascular disease requiring treatment in extremely preterm infants
Source: BMC Pediatr. 2024 Jul 20;24:467. doi: 10.1186/s12887-024-04943-4 (PMC11264936; doi:10.1186/s12887-024-04943-4)
Supplement: Supplementary file 1 — Supplementary Material 1. [file 12887_2024_4943_MOESM1_ESM.docx]

**Supplemental Table 1.** Baseline characteristics of the patients included in this study.

|  | **No PH treatment (n = 90)** | **PH treatment (n = 67)** | **P** |
| --- | --- | --- | --- |
| **Maternal factors** |  |  |  |
| Maternal age, y | 35.0 [32.0;37.0] | 35.0 [32.0;37.0] | 0.839 |
| Oligohydramnios, n (%) | 20 (22.2%) | 22 (32.8%) | 0.192 |
| PROM, n (%) | 53 (58.9%) | 30 (44.8%) | 0.112 |
| PROM duration, d | 6.0 [2.0;17.5] | 23.0 [5.0;42.0] | **0.009** |
| Chorioamnionitis, n (%) | 57 (64.8%) | 31 (46.3%) | **0.018** |
| Antenatal corticosteroids, n (%) | 83 (92.2%) | 63 (94.0%) | 0.760 |
| Cesarean section, n (%) | 49 (54.4%) | 32 (47.8%) | 0.505 |
| Maternal DM, n (%) | GDM 8 (8.9%) Overt DM 4 (4.4%) | GDM 6 (9.0%) Overt DM 2 (3.0%) | 1.000 |
| Maternal hypertensive disorder, n (%) | PIH 4 (4.4%) Chronic HTN 1 (1.1%) | PIH 6 (9.0%) Chronic HTN 5 (7.5%) | 0.057 |
| **Neonatal factor** |  |  |  |
| GA, wk | 26.1 ± 1.2 | 25.2 ± 1.4 | **0.000** |
| Body weight, g | 831 ± 185.3 | 706.3 ± 187.8 | **0.000** |
| Small for gestational age, n (%) | 8 (8.9%) | 10 (14.9%) | 0.312 |
| Multiple gestation, n (%) | Singleton 28 (31.1%) Twin 50 (55.6%) Triplet 12(13.3%) | Singleton 20 (29.4%) Twin 37 (55.2%) Triplet 10 (14.9%) | 0.956 |
| Apgar score 1 min | 3 [2; 5] | 3 [1; 4] | **0.004** |
| Apgar score 5 min | 7 [5;7] | 6 [4;7] | **0.008** |
| **Other outcomes, n(%)** |  |  |  |
| Respiratory distress syndrome, n (%) | 77 (85.6%) | 67 (98.5%) | **0.004** |
| Air leak, n (%) | 2 (2.2%) | 13 (19.4%) | **0.000** |
| Pulmonary hemorrhage, n (%) | 9 (10.0%) | 10 (14.9%) | 0.459 |
| Neonatal seizure, n (%) | 4 (4.4%) | 7 (10.4%) | 0.207 |
| Intraventricular hemorrhage, n (%) | 7 (8.0%) | 14 (22.6%) | **0.016** |
| Congenital infection, n (%) | 2 (2.2%) | 2 (3.0%) | 1.000 |
| Early onset sepsis, n (%) | 6 (6.7%) | 13 (19.4%) | **0.024** |
| Late onset sepsis, n (%) | 16 (17.8%) | 12 (17.9%) | 1.000 |
| Necrotizing enterocolitis, n (%) | 7 (7.8%) | 3 (4.5%) | 0.518 |
| Spontaneous intestinal perforation, n (%) | 7 (7.8%) | 8 (11.9%) | 0.420 |
| Surgical necrotizing enterocolitis, n (%) | 6 (6.7%) | 3 (4.5%) | 0.734 |
| Surgical retinopathy of prematurity, n (%) | 49 (54.4%) | 34 (50.7%) | 0.766 |
| Moderate to large PDA, n (%) | 68 (75.6%) | 46 (68.7%) | 0.437 |
| PDA treatment, n (%) | 60 (66.7%) | 35 (52.2%) | 0.096 |
| PDA ligation operation, n (%) | 25 (27.8%) | 21 (31.3%) | 0.758 |

Data are expressed as numbers (%) or medians [interquartile range] for using nonparametric method, or mean ± SD for using parametric method.

Chronic HTN, Chronic hypertension; DM, diabetes mellitus; GA, gestational age; GDM, gestational diabetes mellitus; PDA, patent ductus arteriosus; PIH, pregnancy-induced hypertension; PROM, premature rupture of membranes; PH, pulmonary hypertension.

Chi-square test and t-test were performed to describe difference for parametric analysis, Fisher’s exact test, and Wilcoxon rank-sum test were performed to describe the differences for nonparametric analysis.

**Supplemental Table 2.** Outcomes of patients included in this study.

|  | **No PH treatment (n = 90)** | **PH treatment (n = 67)** | **P** |
| --- | --- | --- | --- |
| Death at discharge, n (%) | 4 (4.4%) | 29 (43.3%) | **0.000** |
| Duration of admission*, d | 96.5 [81.0;121.0] | 133.5 [103.0;163.0] | **0.000** |
| BPD severity (2019 NICHD-NRN Jensen)*, n (%) | No BPD 43 (50.0%)  Grade 1 19 (22.1%)  Grade 2 21 (24.4%)  Grade 3 3 (3.5%) | No BPD 7 (18.4%)  Grade 1 2 (5.3%)  Grade 2 20 (52.6%)  Grade 3 9 (22.7%) | **0.000** |
| Duration of invasive ventilation*, d | 17.5 [6.0;40.0] | 45.5 [32.0;67.0] | **0.000** |
| Duration of noninvasive ventilation*, d | 37.5 [27.0;50.0] | 45.5 [28.0;65.0] | 0.122 |
| Duration of supplemental oxygen*, d | 75.5 [54.0;117.0] | 157.5 [102.0;261.0] | **0.000** |

Data are expressed as numbers (%) or medians [interquartile range] for using nonparametric method. * Except for infants who died at discharge.

BPD, bronchopulmonary dysplasia; NICHD, Eunice Kennedy Shriver National Institute of Child Health and Human Development; NRN, Neonatal Research Network; PH, pulmonary hypertension.

Fisher’s exact test and Wilcoxon rank-sum test were performed to describe the differences for nonparametric analysis.

**Supplemental Table 3.** Deceased patients’ characteristics in this study groups.

|  | **Extremely early-period treatment (n = 21)** | **Early-period treatment (n = 8)** |
| --- | --- | --- |
| **Maternal factors** |  |  |
| Maternal age, y | 34.6 ± 3.6 | 33.9 ± 2.6 |
| Oligohydramnios, n (%) | 11 (52.4%) | 1 (12.5%) |
| PROM, n (%) | 9 (42.9%) | 2 (25.0%) |
| PROM duration, d | 37.6 ± 23.8 | 16.5 ± 20.5 |
| Chorioamnionitis, n (%) | 7 (33.3%) | 3 (37.5%) |
| Antenatal corticosteroids, n (%) | 20 (95.2%) | 6 (75.0%) |
| Cesarean section, n (%) | 12 (57.1%) | 2 (25.0%) |
| Maternal DM, n (%) | GDM 1 (4.8%) Overt DM 1 (4.8%) | GDM 0 (0%) Overt DM 0 (0%) |
| Maternal hypertensive disorder, n (%) | PIH 2 (9.5%) Chronic HTN 2 (9.5%) | PIH 1 (12.5%) Chronic HTN 0 (0%) |
| **Neonatal factor** |  |  |
| GA, wk | 25.2 ± 1.5 | 24.5 ± 1.2 |
| Body weight, g | 682.4 ± 226.5 | 626.2 ± 201.9 |
| Small for gestational age, n (%) | 7 (33.3%) | 1 (12.5%) |
| Multiple gestation, n (%) | Singleton 5 (23.8%) Twin 14 (66.7%) Triplet 2 (9.5%) | Singleton 2 (25.0%) Twin 5 (62.5%) Triplet 1 (12.5%) |
| Apgar score 1 min | 2.3 ± 1.4 | 3.6 ± 2.2 |
| Apgar score 5 min | 4.9 ± 2.3 | 6.0 ± 2.4 |
| **Cause of Death, n(%)** |  |  |
| Air leak, n (%) | 2 (9.5%) | 0 (0%) |
| Early onset sepsis, n (%) | 3 (14.3%) | 1 (12.5%) |
| Late onset sepsis, n (%) | 1 (4.8%) | 1 (12.5%) |
| Necrotizing enterocolitis, n (%) | 0 (0%) | 1 (12.5%) |
| PH, n (%) | 10 (47.6%) | 3 (37.5%) |
| Pulmonary hemorrhage, n (%) | 1 (4.8%) | 0 (0%) |
| Pulmonary hypoplasia, n (%) | 3 (14.3%) | 0 (0%) |
| Spontaneous intestinal perforation, n (%) | 0 (0%) | 1 (12.5%) |
| Unknown, n (%) | 1 (4.8%) | 1 (12.5%) |

Data are expressed as numbers (%) or mean ± SD for using parametric method.

Chronic HTN, Chronic hypertension; DM, diabetes mellitus; GA, gestational age; GDM, gestational diabetes mellitus; PIH, pregnancy-induced hypertension; PROM, premature rupture of membranes; PH, pulmonary hypertension


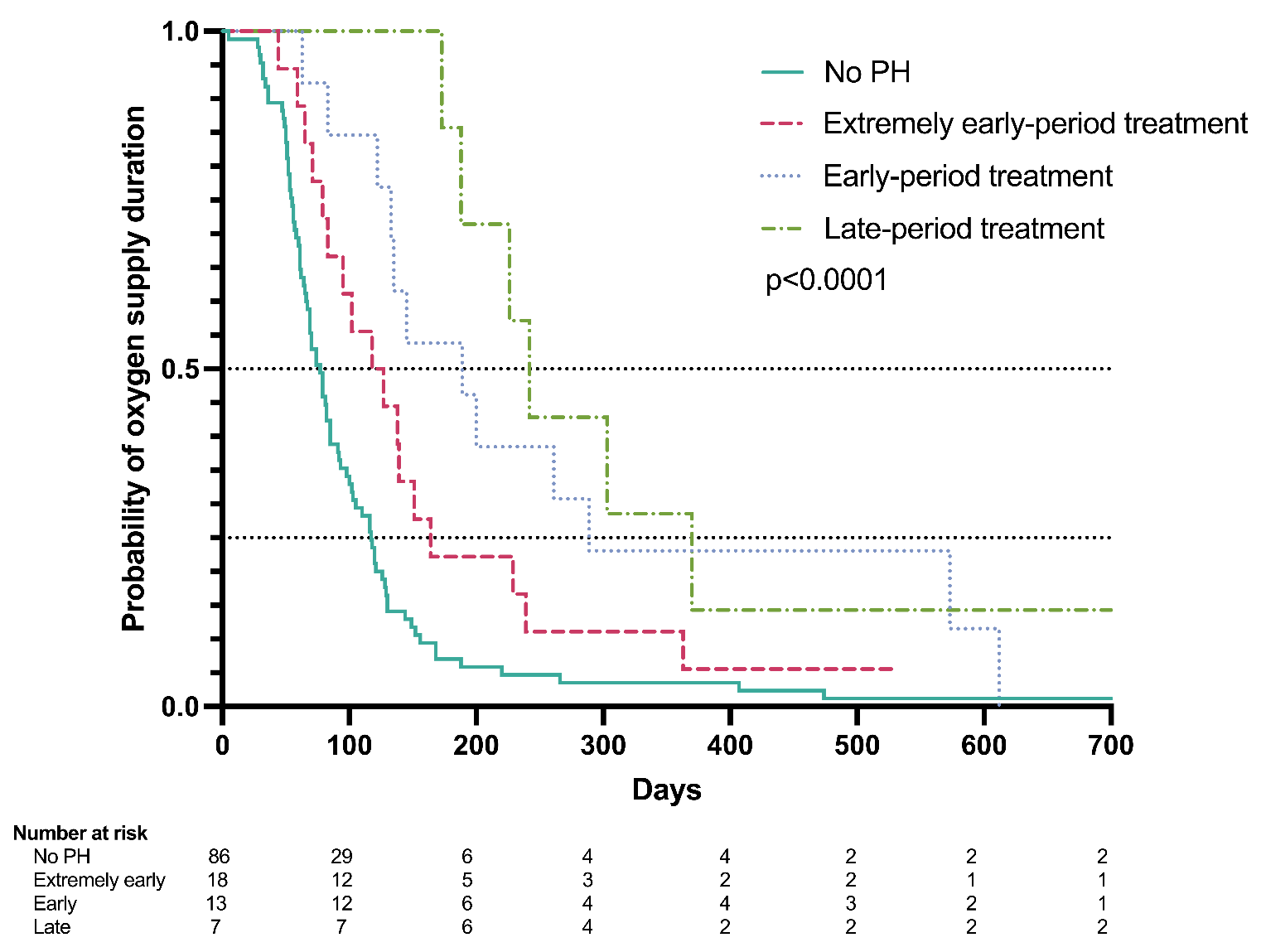


Supplemental Figure 1. Kaplan-Meier curve of supplemental oxygen duration by the initial treatment period of PH.

PH, pulmonary hypertension.

A log-rank test was performed to determine differences.
